# Antithrombotic Therapy in Patients Undergoing Transcatheter Aortic Valve Implantation

**DOI:** 10.3390/jcm13133636

**Published:** 2024-06-21

**Authors:** Francesco Pallante, Francesco Costa, Victoria Garcia Ruiz, Giampiero Vizzari, Pietro Iannello, Lucio Teresi, Gabriele Carciotto, Stefania Lo Giudice, Giustina Iuvara, Giulia Laterra, Ander Regueiro, Gennaro Giustino, Juan Horacio Alonso Briales, Jose Maria Hernandez, Marco Barbanti, Antonio Micari, Francesco Patanè

**Affiliations:** 1 Department of Clinical and Experimental Medicine, University of Messina, 98122 Messina, Italy; francescopallante98@gmail.com (F.P.); giampiero.vizzari@polime.it (G.V.); lucioteresi@gmail.com (L.T.); gcarciotto97@gmail.com (G.C.); stefania.logiudice1996@gmail.com (S.L.G.); giustina.iuvara@gmail.com (G.I.); 2 Department of Biomedical and Dental Sciences and of Morphological and Functional Images, University of Messina, 98122 Messina, Italy; amicari@unime.it (A.M.); fpatane@unime.it (F.P.); 3 Departamento de Medicina UMA, Área del Corazón, Hospital Universitario Virgen de la Victoria, CIBERCV, IBIMA Plataforma BIONAND, 29010 Malaga, Spain; mavigaru@gmail.com (V.G.R.); juanhalonso62@gmail.com (J.H.A.B.); josemaria2509@gmail.com (J.M.H.); 4 Cardiology Division, Papardo Hospital, 98158 Messina, Italy; pietro.iannello@aopapardo.it; 5 Faculty of Medicine and Surgery, Università degli Studi di Enna “Kore”, 94100 Enna, Italy; giulia.laterra@unikore.it (G.L.); marco.barbanti@unikore.it (M.B.); 6 Hospital Clinic, Cardiovascular Institute, Institut D’Investigacions Biomèdiques August Pi I Sunyer (IDIBAPS), 08036 Barcelona, Spain; anderregueiro@gmail.com; 7 Icahn School of Medicine at Mount Sinai, New York, NY 10029, USA; g.giustino@hotmail.com

**Keywords:** antithrombotic therapy, aortic valve stenosis, transcatheter aortic valve implantation (TAVI), transcatheter aortic valve replacement (TAVR), bleeding risk, thrombotic risk, tailored therapy, risk scores

## Abstract

Transcatheter aortic valve implantation (TAVI) now represents the mainstay of treatment for severe aortic stenosis. Owing to its exceptional procedural efficacy and safety, TAVI has been extended to include patients at lower surgical risk, thus now encompassing a diverse patient population receiving this treatment. Yet, long-term outcomes also depend on optimal medical therapy for secondary vascular prevention, with antithrombotic therapy serving as the cornerstone. Leveraging data from multiple randomized controlled trials, the current guidelines generally recommend single antithrombotic therapy, with either single antiplatelet therapy (SAPT) or oral anticoagulation (OAC) alone in those patients without or with atrial fibrillation, respectively. Yet, individualization of this pattern, as well as specific case uses, may be needed based on individual patient characteristics and concurrent procedures. This review aims to discuss the evidence supporting antithrombotic treatments in patients treated with TAVI, indications for a standardized treatment, as well as specific considerations for an individualized approach to treatment.

## 1. Rationale for Antithrombotic Therapy after TAVI

**Figure 1:**
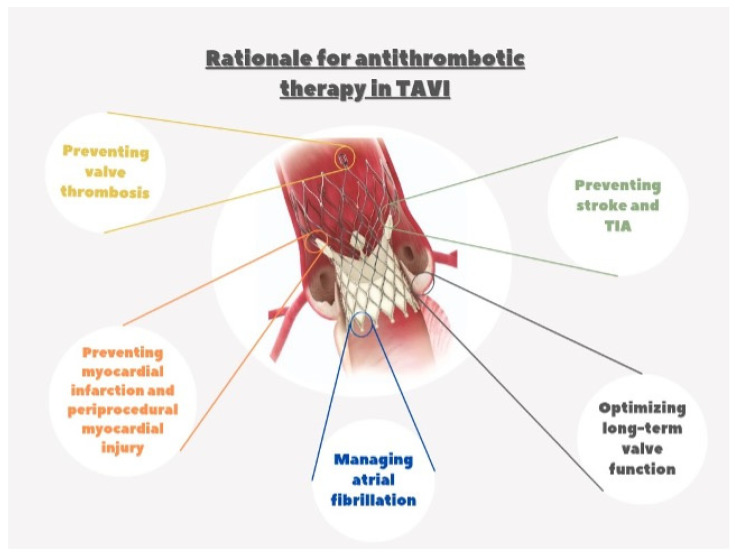
Rationale for antithrombotic therapy in TAVI.

The association between percutaneous cardiovascular intervention (PCI) and antithrombotic therapy dates back to the early days of coronary angioplasty. Since the introduction of this procedure, warfarin anticoagulation after the procedure has been crucial in preventing thrombotic risk [1]. In the subsequent stent era, warfarin alone or with aspirin was initially often prescribed until evidence from randomized trials highlighted lower risks of stent thrombosis with dual antiplatelet therapy (DAPT), which became the mainstay of treatment after stent implantation [2,3,4,5,6]. With the evolution of percutaneous techniques, which included the implantation of dedicated devices for atrial septal defect [7,8], patent foramen ovale (PFO) [9], and leak closures, post-procedural antithrombotic therapy to prevent device thrombosis has been extrapolated from the coronary stent experience, with DAPT used for different periods after the procedure based on scant scientific evidence. The recent development of percutaneous treatment for structural heart disease, including transcatheter aortic valve implantation (TAVI) [10] or transcatheter edge-to-edge mitral valve repair (TMVR) [11] has made no exception, with different DAPT courses used in pivotal approval studies for the first-generation devices. The potential rationale for secondary prevention with antithrombotic therapy after TAVI is multifaced (Figure 1). As previously seen with other intravascular implantable devices, the TAVI metallic frame has the potential to trigger thrombus formation before metallic-strut endothelialization, and this may justify an initial course of antithrombotic therapy [12]. The endothelialization process varies in time and completeness depending on the device size and type [13], and while, theoretically, this may represent a concern for device thrombosis, currently there is no clear evidence that antithrombotic therapy has a true clinical impact [14]. On the other hand, it is well established that after TAVI, patients are exposed to a higher and temporally variable risk of ischemic events, including transient ischemic attack (TIA), stroke, myocardial infarction (MI) [15,16,17,18,19,20,21,22,23,24], and valve thrombosis [25,26]. Hence, assessing the impact of various antithrombotic approaches and valve types [27] to minimize this risk has been the primary focus of numerous clinical studies. In addition, many patients undergoing TAVI have an additional burden of ischemic risk due to concomitant coronary artery disease and coronary stenting, as well as atrial fibrillation with a high thromboembolic risk, which may further justify the need for antiplatelets or oral anticoagulants. Finally, more recent evidence of subclinical valve leaflet thrombosis has triggered interest in preventive antithrombotic strategies to prevent this phenomenon and potentially extend valve durability.

Altogether, while TAVI has transitioned from being cutting edge to the mainstream in recent years, extending the range of patients treated, prospective studies have challenged the concept of DAPT as standard therapy after TAVI and have explored the optimal approach to post-TAVI antithrombotic therapy to optimize its benefits over its risks. On this matter, the potential to reduce therapy-related spontaneous bleeding has been tested and confirmed to have a significant prognostic impact, maintaining similar anti-ischemic efficacy with a minimal antithrombotic therapy. In line with the research that focuses on an individualized treatment approach for patients with coronary artery disease selected for antithrombotic therapy, patients undergoing TAVI have also recently been evaluated for personalized treatment options. In this report, we aimed to examine recent evidence from clinical studies, guidelines, and future perspectives on antithrombotic treatment for patients undergoing TAVI.

## 2. The Initial Experience in the Pivotal Randomized Trials of TAVI

Since the large-scale implementation of TAVI [28], it has become clear how important it is for patients’ prognoses to strike a balance between ischemic and hemorrhagic risk [29]. The early TAVI studies followed antithrombotic protocols largely based on coronary angioplasty guidelines. The FRANCE 2 [30] was a prospective registry that included all TAVI procedures performed in France from 2010 to 2011 with first-generation devices. The patients were treated with both Edward SAPIEN (Edwards Lifesciences LLC, Irvine, CA, USA) and CoreValve (Medtronic, Inc., Minneapolis, Minnesota, USA) devices. All subjects included in the registry received aspirin (≤160 mg/die) and clopidogrel 300 mg during the procedure, and one month of DAPT, followed by aspirin alone afterward.

The U.S. CoreValve [31] trial enrolled patients with severe aortic stenosis considered to be at increased surgical risk, and randomized them to receive either TAVI with the self-expanding [32] transcatheter valve (TAVI group) or a surgical aortic valve replacement (surgical group) from 2011 to 2012. Similarly, DAPT with aspirin and clopidogrel was initiated before TAVI and recommended for 3 months afterward, followed by monotherapy with either aspirin or clopidogrel. In cases of indication of long-term oral anticoagulation, both aspirin and warfarin were given indefinitely.

The PARTNER I trial included two patient cohorts, randomizing patients to either TAVI or surgical aortic valve replacement (SAVR) in patients at high surgical risk [33] (PARTNER 1A), or to TAVI vs. medical therapy in those considered inoperable [34] (PARTNER 1B). The post-procedure antithrombotic protocol included DAPT with aspirin and clopidogrel for 6 months, followed by lifelong aspirin (75 mg to 100 mg daily). No definite recommendations were made for managing periprocedural or subsequent anticoagulation after the TAVI procedure in patients who were already receiving OAC at baseline, and treatment was left to the physicians’ preference. From the onset, it was evident that balancing the risk of thrombosis and hemorrhage was crucial [35].

## 3. Randomized Trials for Different Antithrombotic Therapy Regimens after TAVI

### 3.1. DAPT vs. SAPT

While DAPT for 1 to 6 months after TAVI was the most common treatment strategy in the initial TAVI procedure, subsequent dedicated studies have evaluated the benefits and risks of single antiplatelet therapy (SAPT) after the procedure in patients with no indications for OAC (e.g., atrial fibrillation) (Table 1) [36].

In 2017, the ARTE study [37] was the first to compare DAPT versus SAPT (aspirin) in patients undergoing TAVI and evaluate the efficacy and safety of these strategies. The primary outcomes included death, MI, stroke, TIA, and major or life-threatening bleeding within three months after the procedure, as delineated by the “Valve Academic Research Consortium 2” (VARC-2) [38]. The study was stopped prematurely after enrolling 222 participants (74% of the original cohort) due to slow recruitment and lack of consistent financial support. No significant differences in the primary endpoint were observed at the 90-day follow-up, (15.3% vs. 7.2%, *p* = 0.065), even though events occurred more frequently in the DAPT treatment arm due to an excess of major or life-threatening bleeding with DAPT (10.8% vs. 3.6%, *p* = 0.038).

In 2020, the POPular TAVI [39] cohort A trial compared, in a similar fashion, DAPT vs. SAPT after TAVI. The patients were randomly allocated in a 1:1 ratio to receive aspirin alone or a combination of aspirin and clopidogrel for 3 months post-TAVI. Subsequently, those in the combination group continued with aspirin monotherapy. At 12 months, bleeding from any causes occurred in 15.1% of the SAPT group and 26.6% of the DAPT group (risk ratio, 0.57; 95% confidence interval (CI), 0.42 to 0.77; *p* = 0.001). Similarly, non-procedure-related bleeding occurred in 15.1% and 24.9%, respectively (risk ratio, 0.61; 95% CI, 0.44 to 0.83; *p* = 0.005). Consistently, the secondary composite outcomes of death from cardiovascular causes, non-procedure-related bleeding, stroke from any cause, or MI occurred in 23.0% of patients in the SAPT and 31.1% of those in the DAPT group (95% CI, −14.9 to −1.5; *p* < 0.001). In a recent meta-analysis [40] including four randomized clinical trials comparing aspirin with DAPT, the composite of all-cause mortality, stroke, or MI at 30 days was observed in 5.5% of patients treated with aspirin and in 6.6% of patients treated with DAPT (OR, 0.83; 95% CI, 0.50–1.38, *p* = 0.47), while major or life-threatening bleeding was halved with aspirin alone (5.4% vs. 10.1%, 95% OR, 0.51; CI, 0.32–0.82, *p* = 0.005). In conclusion, multiple studies now support a higher safety and similar efficacy of SAPT after TAVI compared to DAPT.

### 3.2. OAC vs. DAPT

Antithrombotic strategies including OAC after TAVI have been tested in patients both with and without a prior clinical indication for oral anticoagulation, such as atrial fibrillation (Table 1) [41,42,43].

#### 3.2.1. Patients with a Clinical Indication for OAC

Patients undergoing TAVI may have a coexisting clinical indication for anticoagulant therapy [44]. For instance, atrial fibrillation is present in approximately 30–40% of patients undergoing TAVI [31,33,34,45,46,47,48,49,50], and this is particularly relevant because patients undergoing TAVI often have various comorbidities and higher bleeding risk. Although patients undergoing TAVI have a lower incidence of new-onset atrial fibrillation compared to those undergoing SAVR with severe aortic stenosis [51,52], it remains an important element to consider in the management of antithrombotic therapy, especially because it is correlated with an elevated incidence of stroke and mortality during the initial year [24,43,50,53,54,55].

Additional antiplatelet therapy on top of OAC in patients undergoing TAVI has been thoroughly evaluated.

The POPular TAVI cohort B trial [56] compared the effectiveness of post-TAVI therapy with OAC (VKA or a DOAC) and with OAC plus clopidogrel in patients with a clinical indication for OAC, mostly in those with atrial fibrillation. The OAC assigned to patients was based on the specific drug they were utilizing before randomization. At the 12-month follow-up, bleeding according to the VARC 2 definition was observed in 21.7% receiving OAC alone and in 34.6% receiving a combination of OAC plus clopidogrel (risk ratio, 0.63; 95% (CI), 0.43 to 0.90; *p* = 0.01). Non–procedure-related bleeding occurred in 21.7% under OAC alone and in 34.0% with the combination therapy (risk ratio, 0.64; 95% (CI), 0.44 to 0.92; *p* = 0.02). The secondary composite endpoint of death from cardiovascular causes, non–procedure-related bleeding, stroke from any cause, or MI occurred in 31.2% under OAC alone and in 45.5% with the combination of OAC and clopidogrel. These findings suggest that OAC alone may be preferred over combination therapy after TAVI.

The ENVISAGE-TAVI AF [57] tested the efficacy of edoxaban compared with VKA after TAVI. Nearly all patients (99%) enrolled were indicated for OAC for atrial fibrillation before undergoing TAVI. In the intention-to-treat analysis, net adverse clinical events (NACEs) were 17.3% in the edoxaban group and 16.5% in the vitamin K antagonist group. It is worth noting that major bleeding, the study’s primary safety outcome, occurred in 9.7% of the edoxaban group and 7.0% of the vitamin K antagonist group (hazard ratio, 1.40; 95% CI, 1.03 to 1.91; *p* = 0.93 for noninferiority) Hence, while edoxaban demonstrated non-inferiority to VKAs concerning the composite primary efficacy outcome of NACE, non-inferiority for major bleeding was not reached, mainly due to a higher incidence of significant gastrointestinal bleeding in the edoxaban-treated cohort, which may represent an element of concern given the inherent higher risk of gastrointestinal bleeding in patients with severe aortic stenosis.

Another trial testing different types of OAC in TAVI patients was the ATLANTIS trial [58]. Patients were randomized to apixaban at a dose of 5mg bid or to international normalized ratio (INR)-guided VKA therapy throughout follow-up, regardless of the therapy administered before the TAVI. The primary endpoint, a composite of death from any causes, stroke, MI, systemic embolism, intracardiac or valve thrombosis, deep vein thrombosis/pulmonary embolism, or major bleeding, was similar between the two groups (22.0% in the apixaban cohort vs. 21.9% in the VKA cohort, HR 1.02; 0.69–1.51). A similar result was also found for the primary safety endpoints, which included life-threatening, disabling, or major bleeding events defined as Bleeding Academic Research Consortium (BARC) 4, 3a, 3b, and 3c. Additionally, both groups showed similar rates of obstructive valve thrombosis as screened by a four-dimensional computed tomography (4D-CT) scan.

#### 3.2.2. Patients without a Clinical Indication for OAC

In 2015, the seminal observation of the phenomenon of subclinical valve thrombosis sparked interest in the use of OAC after TAVI also in patients without a prior clinical indication for OAC [59]. This investigation examined information from 55 individuals enrolled in a clinical trial (the PORTICO IDE randomized trial) and two single-center registries (the RESOLVE and SAVORY registries), which included 132 patients. The findings revealed that the incidence of diminished leaflet motion was reduced in patients who were administered therapeutic anticoagulation with warfarin during the initial CT scan following TAVI, in contrast to subtherapeutic doses or no anticoagulation (51%). Diminished leaflet motion was also less common among patients undergoing therapeutic anticoagulation (none out of eight patients) than those undergoing DAPT (55%). In the combined RESOLVE and SAVORY registries, reduced leaflet movement was noted in 14% of the enrolled population. Treatment with therapeutic anticoagulation using warfarin demonstrated a reduction of reduced leaflet movement compared to DAPT treatment. These findings suggest that thrombosis is the main determinant of reduced leaflet motion. Moreover, the restoration of leaflet motion with anticoagulation implies that thrombus formation precedes reduced leaflet motion, rather than reduced leaflet motion causing thrombus formation. In addition, in the two combined registries, individuals exhibiting diminished leaflet motion displayed an increased risk of stroke or TIA compared to those with normal leaflet motion. This suggests a potential clinical benefit of preventing these thrombotic complications.

Based on such premises, the GALILEO trial was designed to test whether routine anticoagulation after TAVI might improve clinical outcomes and subclinical valve thrombosis [60]. This study included a total of 1644 patients who were randomized to the OAC group treated with rivaroxaban (10 mg per day) plus aspirin (75–100 mg per day) for 3 months, followed by rivaroxaban monotherapy (10 mg per day), while the antiplatelet-based group was treated with DAPT with aspirin and clopidogrel for 3 months, followed by aspirin monotherapy (75–100 mg per day). The GALILEO trial was interrupted prematurely by the data safety and monitoring board. The primary efficacy endpoint, a composite of death from any cause or thromboembolic events, occurred more frequently among individuals treated with the rivaroxaban-based treatment (12.7%) than in the antiplatelet cohort (9.5%) (HR, 1.35; 95% (CI), 1.01 to 1.81; *p* = 0.04). The primary safety endpoint (composite of life-threatening, disabling, or major bleeding) also trended higher in the rivaroxaban group, despite not reaching formal statistical significance.

These findings brought to light the intricacies of antithrombotic treatment after TAVI. This group typically comprises elderly individuals, who may be frail or have several comorbidities that increase the risks of bleeding and thromboembolic events. Although findings from an imaging subset of GALILEO indicated that rivaroxaban was linked to a reduced incidence of subclinical valve-leaflet thickening and diminished leaflet motion at 90 days compared to antiplatelet therapy, a lack of discernible clinical benefit from rivaroxaban persisted in this particular scenario [61].

Similarly, the ATLANTIS stratum 2 [58] compared apixaban to a short course of DAPT in patients without a clinical indication for anticoagulation after TAVI. The primary outcome, a composite of death, MI, stroke or TIA, non-central nervous system systemic embolism, intracardiac or valve thrombosis, deep vein thrombosis or pulmonary embolism, and life-threatening, disabling, or major bleeding at 1 year, was similar between the two cohorts (16.9% in the apixaban group and 19.3% in the antiplatelet group, HR = 0.88; 95% (CI) (0.66, 1.17); *p* = 0.57). All bleeding outcomes showed comparable results between the apixaban and antiplatelet treatments; nevertheless, an additional signal for higher non-cardiovascular mortality was observed with apixaban. Consistently as in the GALILEO 4D study, a higher rate of obstructive valve thrombosis in the antiplatelet cohort (6.1% vs. 1.1% in the apixaban group (HR 0.19 (0.08–0.46)) was observed.

More recently, the ADAPT-TAVR [62] analyzed the use of edoxaban versus DAPT in preventing leaflet thrombosis [63] and its relationship with cerebrovascular events [26] in individuals without clinical indication for OAC after TAVI. The experimental group was treated with edoxaban (60 mg per day or 30 mg per day) with dose-adjustment criteria. In comparison, the control group received DAPT (aspirin 100 mg per day in combination with clopidogrel at 75 mg per day) for 6 months. The 235 patients enrolled underwent a contrast-enhanced, electrocardiogram-gated cardiac CT scan at the 6-month post-randomization. The 4D-CT scan results showed that 9.8% of the edoxaban cohort had at least one leaflet thrombosis vs. 18.4% of the DAPT cohort (absolute difference of −8.5% (95% CI), −17.8% to 0.8%; *p* = 0.076). Thrombosis of the leaflet with reduced motion grade 3 or higher was observed in 2.9% of the patients treated with edoxaban and in 7.3% of those treated with DAPT. In serial MRI, 25.0% of the patients given edoxaban developed new brain lesions, while 20.2% of the patients given DAPT (risk ratio (95% CI) of 1.24 (0.75 to 2.04), *p* = 0.40). The findings showed that the decrease in leaflet thrombosis did not correlate with the reduction in new cerebral lesions or any onset of neurological or neurocognitive impairment. Furthermore, although hypoattenuated leaflet thickening (HALT) appeared to respond better to OAC than to antiplatelet treatment, as shown in other trials [25,64], there was no detectable link between subclinical leaflet thrombosis and any changes in new cerebral thromboembolic lesions or neurological outcomes occurring concomitantly. However, it should be noted that the study did not have enough statistical power to provide a definitive interpretation for the clinical endpoints.

### 3.3. No Antithrombotic Therapy

While a minimalistic strategy appeared safer in prior studies, a strategy of no antithrombotic therapy after TAVI could, in principle, further improve safety. Kobari et al. [65] evaluated the impact of no antithrombotic therapy after TAVI within the OCEAN-TAVI registry [66]. The study included three groups: the first group of 293 patients did not receive any antithrombotic therapy, the second group of 1354 patients received SAPT (aspirin or clopidogrel), and the third group of 1928 patients received DAPT (aspirin plus clopidogrel). After adjusting for confounding factors, the study revealed no significant differences in the risk of NACEs over the three-year follow-up among the three distinct approaches. In particular, major and life-threatening bleeding was less frequent in the group that did not receive antithrombotic treatment (none, 4.1%; SAPT, 6.5%; DAPT, 8.4%; log-rank *p* = 0.07). A notable trend suggested a lower rate of all-cause mortality in the DAPT group, which may entail incomplete adjustment for confounders. Acknowledging the important limitations given by the observational design, this study represents the first evidence of no-antithrombotic therapy after TAVI, which may become useful, particularly for higher-risk patients. Further validation of these results through additional studies is essential to establish the reliability and generalizability of this strategy and will be evaluated in dedicated trials [67].

### 3.4. Personalized Treatment Based on Individual Patient Risk

Considering the current challenges in optimizing antithrombotic therapy in patients undergoing TAVI, personalized assessment using predictive bleeding scores could be a viable solution to optimize the benefits over the risks of antithrombotic therapy. Navarese et al. developed a novel tool for predicting bleeding complications after TAVI (PREDICT-TAVR score) [68]. PREDICT-TAVR is a scoring system comprising six factors: pre-procedural hemoglobin, serum iron level, common femoral artery diameter, creatinine clearance, DAPT post-TAVI, and OAC. The score has been externally validated in the POL-TAVI database [69], demonstrating decent discrimination of bleeding events at 30 days, with an AUC of 0.78 (95% CI: 0.72–0.82). PREDICT-TAVR showed superior performance in terms of discrimination and reclassification compared to the PARIS and HAS-BLED scores [70,71]. More recently, Yuheng Jia et al. [72] developed a prediction model for late major bleeding after TAVI from a single-center retrospective registry in China. The novel model, BLeNet, was generated using deep learning (DL) [73], a subset of machine learning techniques. This model consists of a total of 56 features that cover fundamental, procedural, and post-procedural characteristics. The model has been internally compared with two other models based on the traditional Cox model and a random forest model, with the DL-based model showing superior performance (BLeNet: 0.81; Cox-PH: 0.72; random survival forest: 0.70). Compared to the PREDICT-TAVR, the BLeNet model demonstrated a similar performance. A study [74] evaluated the effectiveness of the “Academic Research Consortium for High Bleeding Risk” (ARC-HBR) criteria in 787 patients randomized from the SCOPE 2 trial [75]. Individuals were regarded as having HBR [76] if they met at least one major or two minor criteria. The primary endpoint included major or life-threatening bleeding (BARC type 3 or 5) at 12 months. Interestingly, ARC-HBR, which is widely adopted after PCI to gauge hemorrhagic risk, has demonstrated poor utility in the TAVI setting, likely due to the unique characteristics of the TAVI population. Therefore, it is important to establish specific HBR criteria for patients undergoing TAVI, whereas extrapolation from other models developed in the PCI and AF setting should be avoided.

### 3.5. Current Guideline Recommendations: ESC/EACTS and ACC/AHA Guidelines

Several similarities and differences should be noted between the ACC/AHA and ESC/EACTS guidelines [77]. The 2021 ESC/EACTS guidelines (class of recommendation I; level of evidence (LOE) A) [78] recommend performing long-term SAPT (75–100 mg of aspirin per day or 75 mg of clopidogrel per day) after TAVI if there is no clinical indication for OAC. If there is an indication for the use of OAC instead, the guidelines suggest the use of long-term OAC alone (class of recommendation I; LOE B). The recommendation for DAPT with aspirin (75–100 mg per day) and clopidogrel (75 mg per day) post-TAVI is limited to cases of recent coronary stent placement (within 3 months), with the period determined by bleeding risk (1 to 6 months), followed by lifelong SAPT. The ACC/AHA 2020 guidelines [79] recommend using SAPT, preferably aspirin, in TAVI patients without an indication for OAC. Furthermore, according to the 2020 ACC/AHA guidelines, VKA may be used with a target INR of 2.5 for at least 3 months in TAVI patients with a low risk of bleeding and no indication for OAC. When dealing with conditions like atrial fibrillation, venous thromboembolism, or hypercoagulability, those who also need OAC should continue OAC after undergoing TAVI. Both VKA and DOAC (direct oral anticoagulant) can be utilized in this situation, following standard dosing and practice guidelines.

### 3.6. Special Conditions: Patients Undergoing Concomitant Coronary Stent Implantation

Another special situation that may complicate the management of antithrombotic therapy with TAVI regards patients with concomitant coronary stent implantation. Considering the lack of clear scientific evidence, an extrapolation of the common practice of patients undergoing PCI is currently indicated, with personalized treatment choices based on individual risk possibly preferred in this higher-risk population [80]. The 2021 Expert Consensus Document of the Thrombosis Working Group of the ESC and the European Association of Percutaneous Cardiovascular Interventions (EAPCI) [81] provided recommendations on antithrombotic treatment in this setting. For those without an indication for OAC, the consensus recommends contemplating DAPT consisting of aspirin and clopidogrel for a period ranging from 1 to 6 months. The duration is correlated with the patient’s characteristics. As TAVI patients are typically at high risk of bleeding, the recommended period of DAPT is usually 1–3 months for chronic coronary syndrome (CCS) and 3–6 months for acute coronary syndrome (ACS) in the majority of cases [6]. Subsequently, a lifelong regimen of SAPT should be considered, either with aspirin or clopidogrel. If a patient has undergone coronary stenting within the last three months before the TAVI procedure and requires OAC therapy, it is recommended to continue lifelong OAC treatment. In addition, SAPT (such as aspirin or clopidogrel) should be taken for a period of 1 to 6 months.

### 3.7. Future Perspective on Optimization of Antithrombotic Therapy in TAVI Patients

Clear scientific evidence is required to manage antithrombotic therapy in patients undergoing TAVI. Future trials may help optimize therapy tailored to patient characteristics. The primary ongoing trials assessing the antithrombotic strategies in TAVI patients are outlined in Table 2. The POPular PAUSE TAVI will study the most suitable approach for anticoagulation during the TAVI procedure. The trial will compare the impact of pausing versus maintaining OAC perioperatively. It will focus on different thrombotic and hemorrhagic outcomes in patients who have received prior OAC therapy and are undergoing TAVI (NCT04437303). The POPular ATLANTIS will explore CT-guided antithrombotic treatment in contrast to lifelong SAPT following the TAVI procedure in patients without a requirement for anticoagulants (NCT06168370). The rationale for this study is the fact that SAPT with aspirin would expose, especially in certain types of patients, a major hemorrhagic risk without a corresponding and adequate reduction in thrombotic-ischemic events. If patients in the experimental group show signs of subclinical valve thrombosis on 4D-CT three months after TAVI, a switch from SAPT to apixaban will be planned. However, if no signs of subclinical valve thrombosis are observed on 4D-CT at the three-month mark after TAVI, and there is no other indication for antiplatelet therapy, SAPT will be discontinued. In cases where no signs of subclinical valve thrombosis are detected on 4D-CT three months post-TAVI, but there is another indication for antiplatelet therapy, SAPT will be continued. In the ACASA-TAVI study, 360 patients without a clinical indication for OAC will be assigned to receive either apixaban at a dosage of 5 mg twice daily, edoxaban at 60 mg per day, or rivaroxaban at 20 mg per day for 12 months. After this period, they will switch to a daily intake of acetylsalicylic acid at 75 mg indefinitely. Alternatively, they may only permanently receive acetylsalicylic acid at 75 mg daily (NCT05035277). In the ACLO-TAVR trial, after 4 weeks of DAPT after TAVI, 230 patients will be randomized to monotherapy with 100 mg of aspirin or 75 mg of clopidogrel. This study will evaluate the onset of leaflet thrombosis with cardiac CT and transthoracic echocardiography at 3 months post-TAVI (NCT05493657). Finally, the NAPT trial [67] will recruit 360 patients undergoing TAVI who will be randomized to aspirin (75 mg to 100 mg) or no-antithrombotic treatment after the procedure. The study’s primary outcome is a combination of death from any cause, heart attack, stroke, and bleeding.

### 3.8. Future Perspective on Optimization of Antithrombotic Therapy in TAVI Patients: New Drugs

Balancing the competing risk of thrombosis and bleeding is a recurring challenge in TAVI patients. As described above, existing anticoagulant agents target multiple factors implicated in the coagulation process, impacting both physiological hemostasis and pathological thrombosis mechanisms. The lack of specificity contributes to the rise in hemorrhagic complications, consequently impacting patients’ outcomes. The process of human coagulation incorporates 3 stages: initiation, amplification, and propagation, occurring on cellular surfaces. In the initiation phase, a small quantity of thrombin is produced from prothrombin, which is crucial for both hemostasis and thrombosis. During the amplification phase, thrombin activates a restricted number of platelets and other coagulation factors, such as factor XI (FXI), which in turn boosts the downstream activation of factors IX and X. In the propagation phase, there is a burst in thrombin generation, which ultimately converts fibrinogen into fibrin. Since thrombus initiation in hemostasis is relatively independent of FXI activation, the small amount of fibrin produced in the initiation phase allows for the formation of an efficacious hemostatic thrombus. This type of thrombus is frequently self-limiting with no additional growth and propagation. In pathological thrombosis, FXI plays a crucial role in mediating the amplification phase, necessary for the burst and growth of the thrombus within the vessel. Targeting FXI appears to be a promising strategy to separate the pharmacological effects from the adverse events of anticoagulant treatment [82]. In small phase 2 trials, the oral FXI inhibitor asundexian showed lower bleeding rates compared to apixaban in patients with atrial fibrillation (PACIFIC-AF) and no significant increment in bleeding when added to antiplatelet treatment (PACIFIC-STROKE and PACIFIC-MI). Future studies could try to establish whether FXI inhibition may represent an optimal antithrombotic strategy for patients after TAVI, preserving the benefit of anticoagulation therapy on bioprosthetic valve function while minimizing the risk of bleeding events, which could impair patients’ outcomes and quality of life.

## 4. Conclusions

TAVI has progressively become the mainstay of treatment for severe aortic stenosis, extending indications for a diverse spectrum of patients. Together with the development of the technique, several dedicated trials now inform the optimal antithrombotic therapy after the procedure. In this setting, a progressive reduction of treatment intensity, preferring single over dual antiplatelet therapy, and OAC alone over dual therapy with OAC plus antiplatelet therapy, has emerged as the best treatment strategy in the majority of patients. While oral anticoagulation has shown promise regarding better valve function, with reduced risk of leaflet thrombosis, worse clinical outcomes in rigorous randomized trials suggest refraining from using this strategy routinely in patients without an established clinical indication. Future studies may shed light on the ability of even less aggressive strategies as well as novel drugs to optimize the benefits over the risks of antithrombotic treatment in this setting.

## Figures and Tables

**Table 1:** Randomized controlled trials testing antithrombotic treatment strategies in patients treated with transcatheter aortic valve implantation.

Study Title	Patients Enrolled (n)	Target Population	Experimental Treatment	Control Treatment	Primary Endpoint (Experimental Group vs. Control Group)
ARTE	222	Patients undergoing TAVI without an indication for OAC	Aspirin + clopidogrel	Aspirin monotherapy	Composite of death, MI, stroke, TIA, or major/life-threatening bleed (at 90-day follow-up (15.3% vs. 7.2%) (OR 95% (CI) = 2.31 (0.95–5.62); *p* = 0.065)
POPular TAVI cohort A	665	Patients undergoing TAVI without an indication for OAC	Aspirin monotherapy	Aspirin + clopidogrel	Two primary endpoints: All bleeding (minor, major, life-threatening, or disabling) (15.1% vs. 26.6%) (risk ratio, 0.57; 95% (CI), 0.42 to 0.77; *p* = 0.001) Non-procedure-related bleeding (including bleeding at the puncture site) (15.1% vs. 24.9%) (risk ratio, 0.61; 95% (CI), 0.44 to 0.83; *p* = 0.005)
POPular TAVI cohort B	313	Patients undergoing TAVI with an indication for chronic OAC	VKA or DOAC	OAC + clopidogrel	Two primary endpoints: All bleeding (minor, major, life-threatening or disabling) (21.7% vs. 34.6%) (risk ratio, 0.63; 95% confidence interval (CI), 0.43 to 0.90; *p* = 0.01) No-procedure-related bleeding (including bleeding at the puncture site) (21.7% vs. 34.0%) (risk ratio, 0.64; 95% (CI), 0.44 to 0.92; *p* = 0.02)
ATLANTIS stratum 1	451	Patients undergoing TAVI with an indication for chronic OAC	Apixaban	VKA	Composite of death, MI, stroke, or TIA, non–central nervous system embolism, pulmonary embolism, intracardiac or valve thrombosis, deep vein thrombosis, and life-threatening, disabling, or major bleeding (22.0% vs. 21.9%) (HR 1.02; 0.69–1.51; *p* = NS)
ENVISAGE-TAVI AF	1426	Patients undergoing TAVI with an indication for chronic OAC	Edoxaban	VKA	Composite of all-cause death, MI, ischemic stroke, systemic thromboembolism, valve thrombosis, or major bleeding (17.3% vs. 16.5%) (HR, 1.40; 95% (CI), 1.03 to 1.91; *p* = 0.93 for noninferiority)
GALILEO	1644	Patients undergoing TAVI without an indication for OAC	Rivaroxaban + aspirin	Clopidogrel + aspirin	Efficacy outcome: death or thromboembolic event (ie, stroke, MI, symptomatic valve thrombosis, non–central nervous system systemic embolism, pulmonary embolism, or deep vein thrombosis) (12.7% vs. 9.5%) (HR, 1.35; 95% (CI), 1.01 to 1.81; *p* = 0.04) Safety outcome: major, life-threatening, or disabling bleed (5.6% vs. 3.8%) (HR 1.50 (0.95 to 2.37); 95% (CI))
GALILEO 4D	231	Patients undergoing TAVI either native or ViV	Rivaroxaban + aspirin	Clopidogrel + aspirin	≥1 prosthetic leaflet with >50% RLM, detected on 4D-CT imaging (2.1% vs. 10.9%) (difference, −8.8 percentage points; 95% confidence interval (CI), −16.5 to −1.9; *p* = 0.01)
ADAPT-TAVR trial	229	Patients undergoing TAVI without an indication for OAC	Edoxaban	Aspirin + clopidogrel	Incidence of valve leaflet thrombosis detected on 4D-CT imaging (9.8% vs. 18.4%) (absolute difference, −8.5% 95% (CI), −17.8% to 0.8%; *p* = 0.076)
ATLANTIS stratum 2	1049	Patients undergoing TAVI without an indication for OAC	Apixaban	Aspirin and/or clopidogrel	Composite of death, MI, stroke, TIA, non–central nervous system embolism, pulmonary embolism, intracardiac or valve thrombosis, deep vein thrombosis, life-threatening, disabling, or major bleeding (16.9% vs. 19.3%) (HR = 0.88; 95% (CI) (0.66, 1.17); *p* = 0.57)

**Table 2:** Summary of current ongoing clinical studies.

Trial	N#	Test Arm	Control Arm	Duration	Primary Completion Date	Primary Endpoint
AVATAR (NCT02735902)	170	VKA or DOAC (apixaban or edoxaban)	ASA + VKA/DOAC	12 months post-TAVI	2024	Composite of death, stroke, MI, valve thrombosis, and hemorrhage (as defined by VARC 2)
POPular PAUSE TAVI (NCT04437303)	858	Interruption of OAC	Continuation of OAC	30 days post-TAVI	2024	Composite of cardiovascular mortality, stroke, MI, major vascular complications, and major, disabling, and life-threatening bleeding complications at 30 days post-TAVI, as defined by the VARC-2 criteria
POPular ATLANTIS (NCT06168370)	2500	(1) If subclinical valve thrombosis on 4D-CT, switch from SAPT to apixaban. (2) If no signs of subclinical valve thrombosis on 4D-CT, without another indication for antiplatelet therapy, stop their SAPT. (3) If no signs of subclinical valve thrombosis on 4D-CT, with another indication for antiplatelet therapy. continue lifelong SAPT	Lifelong SAPT after TAVI	3 months post-TAVI	2028	Composite of cardiovascular death, ischemic stroke, TIA, MI, systemic embolism, and clinically significant valve thrombosis according to the VARC-3 criteria and composite of type 1-4 bleeding, according to the VARC-3 criteria
ACASA-TAVI (NCT05035277)	360	Apixaban, rivaroxaban, or edoxaban	Aspirin	From 12 months to 10 years	2026	(1) Hypo-attenuated leaflet thickening on cardiac CT after 12 months. (2) VARC-3 bleeding events, MI or stroke, all-cause mortality, cardiac death, aortic valve re-intervention, heart failure hospitalization, major, life-threatening, or disabling bleeding.
ACLO-TAVR (NCT05493657)	230	Clopidogrel	Aspirin	3 months post-TAVI	2024	Incidence of leaflet thrombosis on cardiac CT
NAPT (NCT06007222)	360	Non-antithrombotic therapy	Aspirin	1 year to 3 years post-TAVI	2025	Composite endpoint consisting of all-cause deaths, MI, stroke from any cause, and bleeding from randomization to end of study
